# The Impact of Heat on an Emergency Department in Italy: Attributable Visits among Children, Adults, and the Elderly during the Warm Season

**DOI:** 10.1371/journal.pone.0141054

**Published:** 2015-10-29

**Authors:** Laura Ghirardi, Giulia Bisoffi, Rina Mirandola, Giorgio Ricci, Michela Baccini

**Affiliations:** 1 Department of Statistics, Informatics and Applications ‘‘G. Parenti”, University of Florence, Florence, Italy; 2 Biostatistics Unit, ISPO Cancer Prevention and Research Institute, Florence, Italy; 3 Research Support and Biostatistical Unit, University Hospital Verona, Verona, Italy; 4 Accident and Emergency Department, University Hospital Verona, Verona, Italy; Columbia University, UNITED STATES

## Abstract

**Introduction:**

Recent studies suggest that heat is associated with an increase in the number of ambulance calls and emergency department visits. We investigated the association between heat and daily number of emergency department visits at the University Hospital of Verona during the warm seasons 2011–2012 and we assessed the magnitude of the impact in terms of attributable events, focusing on the role of age and triage codification.

**Materials and methods:**

We used a Poisson model to analyse the association between daily number of visits and daily mean apparent temperature, accounting for air pollution level and seasonality. The analyses were stratified by age group and were performed both on the total number of emergency department visits and on the subsample of high-priority visits. Impact estimates were obtained only for this subsample, using a Monte Carlo approach to account for sampling variability. Number of attributable events and attributable community rate were calculated.

**Results:**

We found a positive and immediate association between event occurrence and mean apparent temperatures exceeding a threshold located around 28–29°C. The estimated percent change in the total number of visits per 1°C increase of exposure above the threshold was equal to 3.75 (90% CI: 3.01; 4.49). Focusing only on high-priority visits, the estimated percent change was larger and the greatest effect was among children. We estimated that apparent temperatures above the threshold were responsible for 1177 high-priority visits during the study period. Due to the record high temperatures observed in 2012 in Italy and in Europe, the impact in 2012 was much larger than in 2011, and consisted in 34 high-priority visits every 10000 children, 30 every 10000 people aged 15–64, and 38 every 10000 people aged 65 and over.

**Discussion:**

Our results indicate that heat affects not only the elderly, but also children and non-elderly adults, stressing the need for developing public health preparedness plans for the entire community.

## Introduction

The effect of high ambient temperatures on morbidity and mortality is a matter of real concern in public health, and many studies from different regions of the world have found an association between extreme heat during the warm season and mortality rate or incidence of various syndromes. Analyses on daily count of deaths have provided consistent evidence supporting the existence of an association with high temperature [1–6] and with heat waves or heat extremes [7–10]. Other studies have addressed the question whether heat exposure increases cause-specific hospitalizations [11–16], in some cases failing in confirming the results observed for mortality, with associations lower in size or not significant [11, 15]. On the one hand, this supports the hypothesis that many heat-related deaths can occur in individuals before they come to medical attention. On the other hand, hospitalization could be a poorer indicator of population health, compared to mortality. Because the number of available hospital beds is usually decided based on logistic and administrative issues and not on health needs alone, the number of hospital admissions might poorly reflect actual population demand for health assistance.

More recently, clear evidence arose that high ambient temperatures or heat waves are associated with an increase of ambulance calls or dispatches [8, 17–20] and of emergency department (ED) visits [14, 8, 21–26]. Compared to hospital admissions, ED visits have the advantage to be less influenced by administrative limits and allow to estimate the extent to which population health is affected by heat, even in small cities or areas, where the daily number of deaths is presumably low. Moreover, these outcomes are particularly appropriate if the aim is to develop rapid surveillance systems [27, 28], with the long-term goal of preventing more severe health consequences. In addition, ED visits seem a more suitable outcome to study the effect of high temperatures on different age subgroups such as children and non-elderly adults whose vulnerability to heat could not clearly arise if mortality was analysed.

The aim of this study was to estimate the increase in daily number of ED visits associated with heat during the warm season and to assess the magnitude of the impact in terms of attributable events. The study was conducted in the University Hospital of Verona, a city in the Veneto region, Northern Italy. The area under study includes the city of Verona and few surrounding municipalities, for a total of 327000 inhabitants. This area is characterized by a humid subtropical climate, typical of Northern Italy's inland plains, with hot summers and cold winters. We investigated the effect of heat by age class and we focused on the association between heat and emergency visits classified as having higher intervention priority.

## Materials and Methods

### Emergency Visits Data

ED visit data were collected for University Hospital of Verona (Verona, Italy) for the period from 15^th^ May to 15^th^ September for years 2011 and 2012. We included in our study only visits of patients living in the city and in neighbouring municipalities, which are part of Verona hospitals service area and fulfilled a priori defined geographical criteria aimed to guarantee exposure homogeneity. We excluded patients living in municipalities located in hill areas, identified according to the definition provided by ISTAT Atlas of statistical and administrative geography [29].

ED records included information about birth date, residential status, discharge diagnosis (ICD-9-CM) and triage code at discharge, i.e. the code, assigned by the ED physician in accordance with the regional decree DGR n. 1868 of 15th November 2011, which classifies patients based on the intervention priority. We considered all visits with exception of those with primary diagnosis at discharge corresponding to: “congenital malformations” (ICD-9 codes 740–759); “certain conditions originating in the perinatal period” (ICD-9 codes 760–779); “complications of pregnancy, childbirth, and the puerperium” (ICD-9 codes 630–677); “factors influencing health status and contact with health services” (ICD-9 codes V01-V85); “injury and poisoning” (ICD-9 codes 800–999).

A sub-group of high-priority visits requiring urgent treatment (“no-white tag visits”) was identified for additional analyses, based on the triage codification. Individual patient tracking information was not available; therefore, our analyses focused on the overall visit rate and did not address repeated visits by the same individuals.

### Environmental Data

We collected daily mean temperatures and daily mean dew point levels measured in Verona during the period from 15^th^ May to 15^th^ September for the years 2011 and 2012 (from the website www.ilmeteo.it). In order to characterize exposure, these measurements were combined to calculate the daily mean apparent temperature, an index of thermal discomfort proposed by Kalkstein and Valimont (1986) [30], frequently used in the literature for heat exposure [2, 6, 31].

In order to adjust for the confounding effect of air pollution, daily mean concentrations of particles up to 10 micrometers in diameter (PM_10_) were collected from two monitoring stations, one located in a sub-urban area and one located in an urban area close to high traffic roads. For each day, a mean of the values from these two monitoring stations was computed and used in the analyses. Data come from BRACE data warehouse, which includes different kind of information about stations and measurement tools used for air quality monitoring (http://www.brace.sinanet.apat.it). The choice of considering PM_10_ as air pollution indicator was based on data availability and completeness, as well as on previous literature [2, 18, 24].

Apparent temperature was missing on only three days. We filled in these missing data by averaging the apparent temperatures in the previous day and in the following one. For 18 days PM_10_ data were available only from one of the monitoring stations; in this case we used the available values instead of the mean. There were no days with missing information from both monitoring stations.

In the absence of information about people’s time activity patterns and of finer spatial detail for exposures, we assumed that all residents were exposed to the same daily levels of apparent temperature and air pollution.

### Statistical analysis

A Generalized Linear Model (GLM) was used to analyze the association between mean apparent temperature and number of ED visits, assumed to follow a Poisson distribution. We adjusted for the confounding effect of air pollution by introducing in the model a linear term for the daily mean levels of PM_10_. Indicators for day of the week and month were also included in order to control for seasonality. The analysis was stratified by age (0–14; 15–64; 65+).

Heat exposure was defined as the mean apparent temperature at lag 0–3, i.e. the average of mean apparent temperature in the current day and in the previous three days. This indicator is reasonably sensitive to short-term but possibly not immediate effects of exposure on outcome. In order to describe the relationship between temperature and number of visits, a flexible parametric approach was adopted, by modelling the heat-response function through a cubic regression spline with 4 degrees of freedom [2, 18]. Then, the heat-response function was described using two linear terms constrained to join at a common point, or threshold, which was estimated using the maximum likelihood approach [32]. Next, we focused on the association above the threshold. All the analyses were performed on the total number of visits and on no-white tag ED visits.

In order to evaluate robustness of results to the presence of intra-summer correlation (not allowed by GLM), we adopted a Generalized Estimating Equation (GEE) approach, defining exchangeable or first order autoregressive correlation structure [2]. Moreover, the effect of current apparent temperature (lag 0) and the cumulative effect at lag 0–5 were estimated, in order to check sensitivity of results to different exposure specifications focused on the immediate or on the shortly lagged effect of heat, respectively. The choice of inspecting lag 0 to 5 was based on previous studies evaluating ambulance calls and attendances and ED visits, which found evidence of an immediate or shortly delayed effect of heat on these health outcomes [8, 18, 19].

Impact estimates were obtained only for no-white tag ED visits and quantified in terms of number of events attributable to apparent temperatures exceeding the threshold in each class of age during the study period [33, 34]. We used a Monte Carlo approach to obtain, for each class of age, a whole distribution of the attributable events, so that sampling variability around threshold and slope was accounted for [33–35]. First, 10000 values were sampled from the distribution of the slope (b_i_, i = 1,2…10000) and from the distribution of the threshold (h_i_, i = 1,2…10000) assumed to be independent and normally distributed. Then, for each sampled pair (b_i_ h_i_), a daily time series of attributable ED visits (AV) by age group was calculated, according to the following formula:

$$AV_{it}=Y_{t}-Y_{t}\times \text{exp}\left[-b_{i}\left(T_{t}-h_{i}\right)\right]\;if\;T_{t}>h_{i}\;\;\;\;\;\;\;\;\;\;\;\;\;\;AV_{it}=0\;if\;T_{t}\leq h_{i}$$

where T_t_ and Y_t_ are the observed daily mean apparent temperature at lag 0–3 and the observed daily number of ED visits at time t, respectively. Finally, the distribution of the total number of attributable events during the study period and in each warm season was obtained by summing AV_it_ over t, for each i. This distribution was summarized by its median and its 10^th^ and 90^th^ percentiles.

The attributable community rate (ACR) was calculated, separately for the two years, as the ratio between number of attributable events and number of inhabitants of the study area. ACR represents the event rate during summer due to heat exposure in the population [36]. We calculated also the expected number of attributable events per day above the apparent temperature threshold and the maximum daily number of attributable events.

All analyses were conducted using Stata/SE 12 (StataCorp, College Station, TX) and R Software 3.1.2 (Team R Development Core).

This study was approved by Ethics Committee of the University Hospital Verona, Verona, Italy.

## Results

From 15^th^ May 2011 to 15^th^ September 2011, 16850 visits, which satisfied the inclusion criteria explained above, were observed, with an average of almost 136 visits per day. Within the same period, in 2012, there were 20222 visits, approximately 163 per day. Most of visits were required by individuals aged 15–64 (50.8%). The percentage of no-white tag visits over the total number of ED visits in the younger class of age was 50.4%; this percentage was much higher in the other classes of age (80.8% for individuals aged 15–64 and 90.6% for individuals aged 65 and over) (Table 1).

**Table 1:** Estimated effect of mean apparent temperature above the threshold on ED visits at lag 0–3.

Outcome	Age class	Number of visits during the study period	Percent change above the threshold	90% CI	Chi-square test for interaction (2 degrees of freedom)	p-value
**All ED visits**	**All ages**	37072	3.75	(3.01; 4.49)		
	**0–14**	5738	2.31	(0.50; 4.15)	5.95	0.051
	**15–64**	18827	4.53	(3.60; 5.46)		
	**65+**	12507	3.22	(2.14; 4.32)		
**No-white tag visits**	**All ages**	29445	5.06	(4.19; 5.95)		
	**0–14**	2895	6.80	(4.17; 9.49)	7.33	0.026
	**15–64**	15213	5.74	(4.641; 6.84)		
	**65+**	11337	3.70	(2.47; 4.93)		

Point estimates and 90% confidence intervals (90% CI) for the percent changes in the number of ED visits associated to 1°C increase in mean apparent temperature above the threshold at lag 0–3, for all ages and by class of age (0–14, 15–64, 65+); results for all ED visits and for the subset of no-white tag ED visits.

The average daily mean apparent temperature during the warm season was equal to 24.7°C in 2011 and equal to 27.3°C in 2012. The average daily concentration of PM_10_ was equal to 26.5 μg/m^3^ in 2011 and equal to 24.3 μg/m^3^ in 2012.

**Fig 1:**
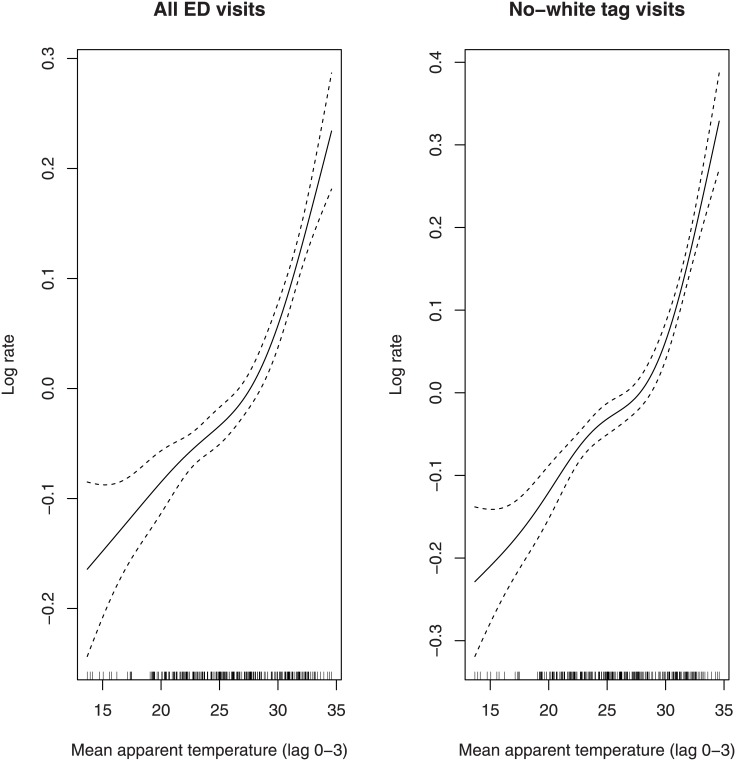
Estimated heat-response curves and 90% confidence bands for all the ED visits and for no-white tag visits at lag 0–3. The maximum likelihood approach provided stable estimates of the threshold even when using different starting points for the algorithm [32]. When considering all ED visits, the threshold was equal to 28.6°C (90% CI: 28.5°C; 28.7°C). When considering the subgroup of no white tag visits, the threshold did not change: the estimated threshold was equal to 28.81°C (90% CI: 28.7°C; 28.9°C).

The heat-response curves is displayed in Fig 1; the relationship was not far from linearity, but a certain evidence arose that the association was stronger for mean apparent temperature above a threshold located around 28–29°C. Using 3 or 5 degrees of freedom did not affect the shape of the relationship (results not shown).

Table 1 reports the results concerning the effect of heat above the threshold; 90% confidence intervals (CI) are reported, instead of the more usual 95% CIs, in order to discourage the use of confidence intervals as hypothesis tests with alpha = 0.05 [37–38]. The overall estimated percent change in the number of ED visits per 1°C increase of mean apparent temperature above the threshold was equal to 3.75 (90% CI: 3.01; 4.49). When the analysis was conducted on the subgroup of no-white tag visits, the percent change was equal to 5.06 (90% CI: 4.19; 5.95).

Introducing in the model the interaction between age class and exposure above the threshold, we found that the slope above the threshold was higher among individuals aged 15–64, compared to younger and older individuals when considering all visits. We estimated that, within this age class, visits increased by 4.53% (90% CI: 3.60; 5.46) per 1°C increase of exposure above the threshold. Smaller increments were estimated for the elderly, 3.22 (90% CI: 2.14; 4.32), and for children, 2.31 (90% CI: 0.50; 4.15). On the contrary, when the focus was on the subgroup of no-white tags, the strongest association was found among children: 6.80 (90% CI: 4.17; 9.49).

Sensitivity analyses considering mean apparent temperature at lag 0–5 did not bring to different results. A slightly smaller effect was found at lag 0. Complete results are reported in Table 2. Analyses using GEE approach and assuming different autocorrelation structures within year did not lead to substantially different results (not reported).

**Table 2:** Estimated effect of mean apparent temperature above the threshold on ED visits at lag 0 and lag 0–5.

Outcome	Exposure—mean above threshold apparent temperature	Age class	Percent change above the threshold	90% CI	Chi-square test for interaction (2 degrees of freedom)	p-value
**All ED visits**	**Lag 0**	**All ages**	2.96	(2.36; 3.56)		
		**0–14**	1.72	(0.22; 3.23)	5.59	0.061
		**15–64**	3.57	(2.81; 4.33)		
		**65+**	2.60	(1.71; 3.50)		
	**Lag 0–5**	**All ages**	3.94	(3.15; 4.74)		
		**0–14**	2.76	(1.14; 4.41)	3.65	0.1615
		**15–64**	4.60	(3.60; 5.60)		
		**65+**	3.49	(2.32; 4.66)		
**No-white tag visits**	**Lag 0**	**All ages**	4.02	(3.33; 4.71)		
		**0–14**	5.17	(3.04; 7.33)	5.21	0.074
		**15–64**	4.49	(3.63; 5.37)		
		**65+**	3.08	(2.10; 4.07)		
	**Lag 0–5**	**All ages**	5.04	(4.10; 5.99)		
		**0–14**	7.75	(5.38; 10.18)	8.26	0.0161
		**15–64**	5.55	(4.37; 6.73)		
		**65+**	3.64	(2.34; 4.97)		

Point estimates and 90% confidence intervals (90% CI) for the percent changes in the number of ED visits associated to 1°C increase in mean apparent temperature above the threshold at lag 0 and at the lag 0–5, for all ages and by class of age (0–14, 15–64, 65+); results for all ED visits and for the subset of no-white tag ED visits.

Estimated impacts are shown in Tables 3 and 4. The days with apparent temperature above the threshold of 28.8°C were almost 30% of the total number of days in the period under study, namely 17 during 2011 and 57 during 2012. We estimated that apparent temperatures above the threshold were responsible in 2011 of 11 high-priority visits among children (10^th^ and 90^th^ percentile: 5; 21), 76 (39; 130), among individuals aged 15–64 and 46 (23; 80) among the elderly, corresponding to an overall ACR of 4.2 high-priority visits every 10000 people. The impact in 2012 was much larger, with 143 (89; 206) attributable high-priority visits among children, 615 (403; 844) among individuals aged 15–64, and 280 (168; 413) among the elderly, corresponding to 33.7 high-priority visits every 10000 children, 30.5 every 10000 people aged 15–64 and 38.1 every 10000 people aged 65 and over. In order to quantify heat impact on ED activity during days with exposure above the threshold, we calculated the average and the maximum daily number of high-priority visits attributable to heat during these days. During 2011, there were on average 7.8 high-priority visits due to heat per day with apparent temperature above the threshold. In 2012 the impact per day was of 18.2 visits: 2.5 among children, 10.8 among non-elderly adults and 4.9 among the elderly. During the same year, we estimated a maximum of 40 daily high-priority visits attributable to heat.

**Table 3:** Estimated impact of apparent temperature above the threshold on no-white tag visits.

	Attributable visits					
	Year					
	2011		2012		2011–2012	
Age class	Median	10^th^ and 90^th^ percentiles	Median	10^th^ and 90^th^ percentiles	Median	10^th^ and 90^th^ percentiles
**All ages**	134	(69, 228)	1042	(690, 1426)	1177	(759, 1652)
**0–14**	11	(5, 21)	143	(89, 206)	155	(95, 227)
**15–64**	76	(39, 130)	615	(403, 844)	692	(442, 974)
**65+**	46	(23, 80)	280	(168, 413)	326	(191, 493)

Median and 10^th^ and 90^th^ percentiles of the distribution of the number of no-white tag visits attributable to daily apparent temperatures above the threshold in 2011, 2012 and total. All results refer to lag 0–3 exposure.

**Table 4:** Attributable community rates by year, expected number of attributable no-white tag visits per day with apparent temperature above the threshold and maximum daily number of attributable no-white tag visits.

		ACR		Expected number of attributable visits per day above the threshold		Maximum number of daily attributable visits	
Age class	Number of inhabitants	Year		Year		Year	
		2011	2012	2011 (17 days above the threshold)	2012 (57 days above the threshold)	2011	2012
**All ages**	317658	4.2	32.8	7.8	18.2	19.8	39.9
**0–14**	42493	2.7	33.7	0.6	2.5	1.6	7.2
**15–64**	201624	3.8	30.5	4.5	10.8	12.3	25.2
**65+**	73541	6.2	38.1	2.7	4.9	6.1	10.6

Number of inhabitants in the area under study at the beginning of 2011; median of the attributable community rate (ACR) distribution in 2011 and 2012; expected number of attributable events per day with apparent temperature above the threshold; maximum daily number of attributable no-white tag visits. All results refer to lag 0–3 exposure.

**Fig 2:**
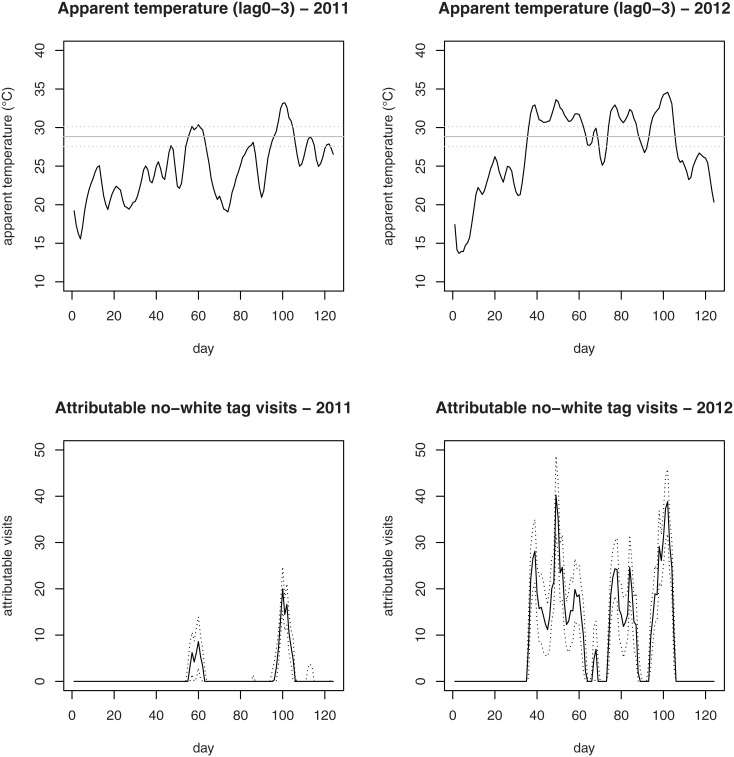
Daily mean apparent temperatures at lag 0–3 and expected daily numbers of attributable no-white tag visits during the study period. Daily mean apparent temperatures at lag 0–3 in 2011 and 2012 (upper panel); expected daily numbers of attributable no-white tag visits (for each day the median of the distribution is reported) in 2011 and 2012 (lower panel). In the upper panel, horizontal continuous lines represent the estimated threshold and dotted lines represent the associated 90% confidence interval (CI); in the lower panel, dotted lines represent the 10th and 90th percentiles of the distribution of the daily number of attributable visits. The values on the *x* axis refer to the calendar days within each warm season, from 1, representing May 15^th^, to 122, representing September 15^th^.

Fig 2 shows the daily mean apparent temperatures at lag 0–3 (upper panel) and the expected daily number of attributable no-white tag visits (lower panel) during 2011 and 2012.

## Conclusions

Studies on the association between heat and ED visits are not as common as studies on mortality. Most of existing literature on this topic has analysed the effect of specific heat-wave episodes [8, 14, 24], with less attention to the role of moderate-to-high temperatures, which occur as a rule during the warm season. Other studies have focused on specific diagnoses or categories of diseases [21–23]. In addition, at the best of our knowledge, health impact analyses have usually evaluated mortality as main outcome [8, 33, 39–40], while only one previous study assessed the burden of ED visits attributable to heat-waves [8].

Overall, our results suggest the existence of a positive association between high apparent temperature and number of ED visits in Verona during the warm seasons of 2011 and 2012. The heat-response function showed a quasi-linear increase, but we found evidence of a steeper slope after a threshold located around 28–29°C. This result is in line with what reported by Alessandrini and colleagues [18] regarding the association between heat and ambulance dispatches in a neighbouring region of Italy.

In our study, the strength of the association varied with age. The percent variations estimated for individuals aged 15–64 were larger than those estimated for older patients. On the one hand, these results are similar to those reported by a recent study on ED visits in Shanghai, which showed a higher effect of heat on people aged <45 years compared to other age groups [25]. On the other hand, we would have expected a larger association among the elderly, according to evidence arising from studies conducted in Italian and European cities, which suggest that older people are frailer and more affected by heat than younger ones, both when analysing mortality [2, 10], ambulance dispatches [18] and ED visits [14, 24, 27 and 28]. The lower effect observed in older people could be due to several factors, which deserve to be better explored. First, the exclusion of traumatic injuries could have led to an underestimation of the effect of heat on the elderly, who could reasonably be more vulnerable, compared to the younger adults, to falls and fractures due to dizziness or syncope related to intense heat. A second plausible reason is related to the fact that the elderly could be more protected from outdoor warmth because less involved in daily activities implying direct heat exposure (e.g. going to school or work). In this sense, our exposure indicator, which is based on outdoor measures of temperature and humidity, could be not able to capture indoor exposure. A third explanation relies on the fact that emergency visits could not be the optimal outcome when evaluating the effects of high temperatures on the elderly, since intense heat could directly lead to premature death, especially among individuals with pre-existing chronic diseases, or with limited ability to take care of themselves or leave home daily [41]. Previous studies, providing evidence that the effect of heat is larger on mortality than on hospital admissions, seem to confirm that mortality could be a more sensitive outcome when measuring the effect of high temperatures in specific subgroups of population [11, 15].

Another important result was the large excess of high-priority visits among children. This strong association did not arise when focusing on all visits. Remarkably, among children white tags were more frequent than in other age groups (50% in children groups vs 30% in individuals aged 15–64 and 20% in older people). This is probably due to the widespread use of emergency departments as a more rapid way to be admitted to a paediatric visit, even for less severe situations. It seems that the exclusion of white tags enabled a better classification of the outcome of interest, allowing detection of the effect of intense heat in children, whose vulnerability could be due to a less efficient thermoregulatory response both for physiological and behavioural reasons [42]. Accordingly, in a recent study a positive association emerged between extreme temperatures and paediatric ED visits for a number of conditions [43]. The effect of heat on children’s health would have probably been missed if hospital admissions or deaths were considered, as suggested by the discrepancies in available evidence about the relationship between heat waves and children’s health [44].

Sensitivity analyses, aimed at estimating the effect of exposure at different lags, did not lead to contradictory results and confirmed the trends found by the main analyses, suggesting that exposure at lag 0–3 is an adequate indicator to capture the short-term effect of heat.

We calculated that in Verona, during the study period, heat was responsible of a not negligible number of no-white tag visits among all age classes. Moreover, this analysis highlighted large heterogeneity of impact between the two warm seasons considered. Remarkably, impact in 2012 was approximately 13% greater than the impact in 2011. This result is explained by the fact that during the warm season of 2012, record high temperatures were registered in Italy and all over Europe (see S1 Fig). Since the number of days above the threshold in 2012 was much larger than in 2011 (57 vs 17), we evaluated also the average number of high-priority visits per day above the threshold, which is a useful indicator of the daily burden weighing down ED in the two years. We estimated that the average number of events per day above the threshold in 2012 was twice the average number in 2011, suggesting the need for developing appropriate plans to face increases in the number of high-priority ED visits under specific meteorological conditions, as in the case of extremely warm summers.

A larger ACR was found for the elderly in both years, despite the smallest effect of heat on ED visits occurrence in this age group. This result underlines the importance of impact assessment, which, combining effect measures with baseline event rates and levels of exposure, contextualizes the effect estimates, providing crucial information for evaluating societal and economic costs and designing prevention plans.

Limits of our study include the fact that only two years were considered (due to administrative issues data older than 2011 were not considered reliable), which makes our results poorly generalizable to other warm seasons. Moreover, we did not investigate the effect of heat on cause-specific ED visits, which theoretically could have provided interesting indications concerning the relationship between heat and specific health events, such as heat-related illness, or respiratory and cardiovascular problems [21–23]. This is in part due to the fact that in our data set the diagnosis codes for ED visits were not highly detailed. Moreover, considering subgroups of visits would have brought to insufficient sample size for investigating how association varies with age and according to intervention priority, which were, on the contrary, our main objectives. Finally, we did not account for additional effect of heat waves, so we likely underestimated the actual association between heat and ED visits, and the related impact. However, in daily mortality analyses, not considering heat waves was found to produce small underestimation of the overall summertime burden of heat, especially when lagged exposures were considered [1]. This result could be valid for different outcomes such as ED visits.

Despite these limitations, our study shows that analysing ED records can be a good option for rapid surveillance of heat effect on population health and can contribute to emphasize the impact of high temperatures also on children and non-elderly adults, for whom the effect of heat could be less evident when measured in terms of mortality excesses.

## Supporting Information

S1 Fig Average of daily mean apparent temperatures during the warm seasons 2003–2013. (EPS)

S1 Data Dataset. The dataset contains information about warm seasons 2011 and 2012, for a total of 248 days. There are 3 rows for each day, corresponding to the three age classes (0–14, 15–64, > = 65). The following variables are reported: daily number of ED visits (*visits*), daily number of no-white tag visits (*priority*), mean apparent temperature in the current day and in the previous three days (*tempapp*, *tempapp_l1*, *tempapp_l2*, *tempapp_l3*), average of these three variables (*tempapp_l03*), level of PM10 in the current day (*PM10_mean*).
(DTA)
